# Supplementary material for: Activation of Autophagic Flux Maintains Mitochondrial Homeostasis during Cardiac Ischemia/Reperfusion Injury
Source: Cells. 2022 Jul 4;11(13):2111. doi: 10.3390/cells11132111 (PMC9265292; doi:10.3390/cells11132111)
Supplement: Supplementary file 1 [file cells-11-02111-s001.zip › cells-1761702-supplementary.pdf]

Supplemental Figures:

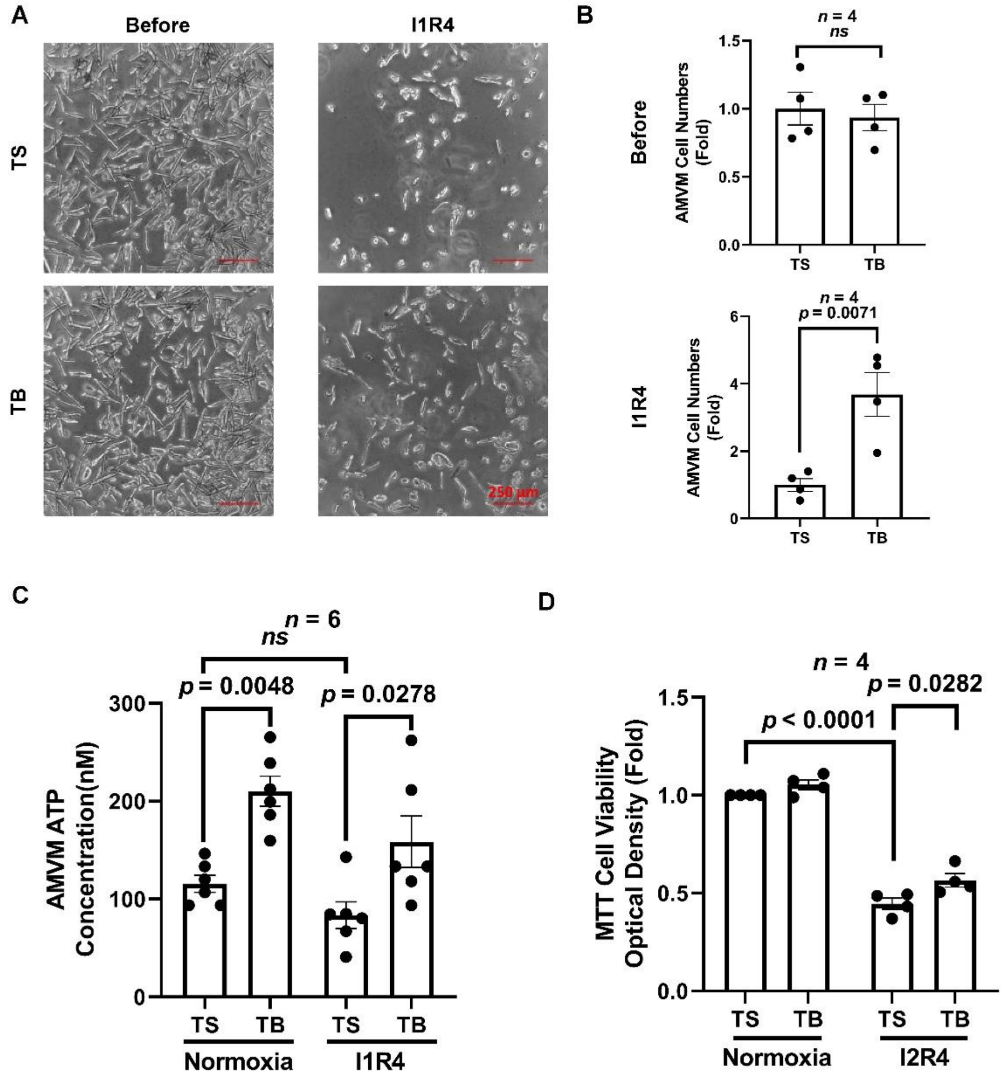

Figure S1. TB reduces cell death in both adult mouse cardiomyocytes and neonatal rat ventricular cardiomyocytes. A. Representative microscopic picture of adult mouse cardiomyocytes. Bar = 250 μ m. B. Quantification of cell number before and after I/R. Cell numbers after I/R, n = 4, TS vs. TB, *p* = 0.0071. C. Cellular ATP was measured in AMVMs. n = 6. Normoxia, TS vs. TB, *p* = 0.0048. I1R4, TS vs. TB, *p* = 0.0278. D. MTT cell viability assay of neonatal rat ventricular cardiomyocytes. MTT cell viability after I/R, n = 4, TS vs. TB, *p* = 0.028. TS, Tat-Scrambled; TB, Tat-Beclin 1; I1R4, Ischemia 1 hour and reperfusion 4 hours; I2R4, Ischemia 2 hours and reperfusion 4 hours; NS (not significant).

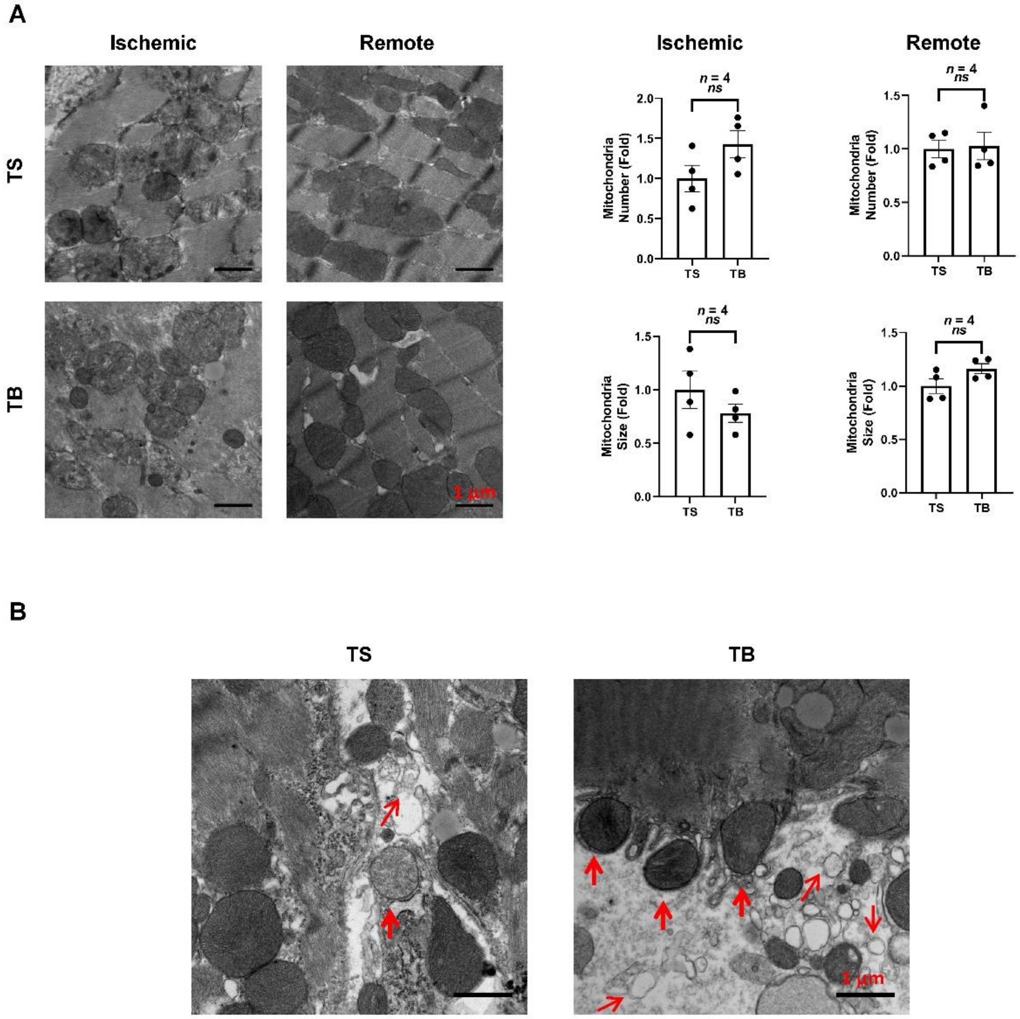

Figure S2. TB treatment does not affect mitochondrial morphology in mouse ischemic and remote zones, but increases autophagosome in the border zone. A. Representative electron microscopic picture of mouse myocardium in the ischemic and remote zones. Bar = 1μm. n = 4, TS vs. TB, NS (not significant). B. Representative electron microscopic picture of mouse myocardium in the border zone (between sarcomeres). Bar = 1μm. Arrows, autophagosome with double membrane enclosing. Some of the mitochondria have been enclosed, suggesting increased mitophagy. TS, Tat-Scrambled; TB, Tat-Beclin 1.

A

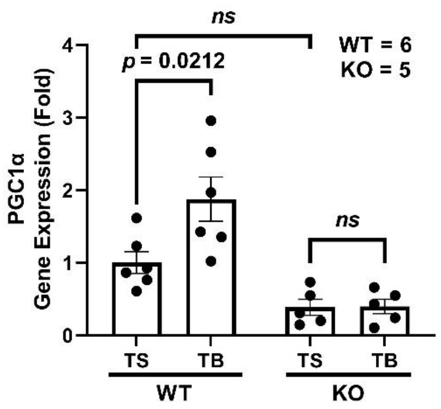

B

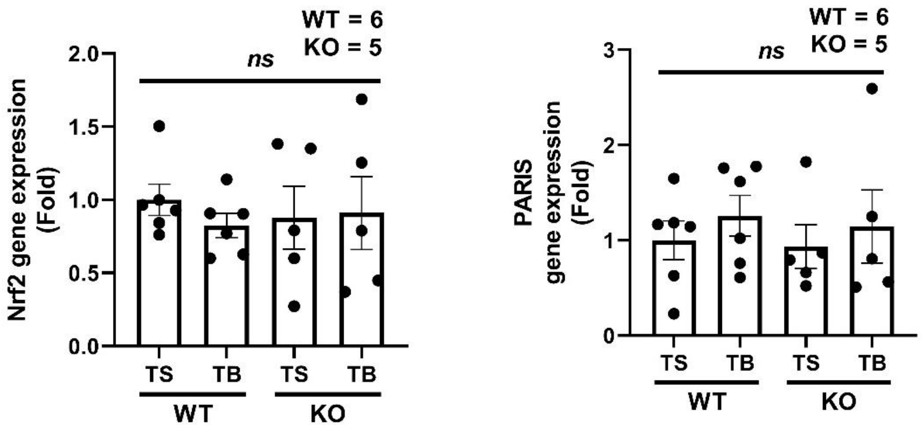

Figure S3. TB treatment increases PGC1α gene expression in AMVMs, but does not increase the expression of two upstream regulators, NRF2 and PARIS. A, PGC1α gene expression in AMVMs. n = 5-6. TS vs. TB, WT, $p = 0.0212$. KO, p = NS (not significant). B, NRF2 and PARIS gene expression in AMVMs. n = 5-6. p = NS. TS, Tat-Scrambled; TB, Tat-Beclin 1; NS (not significant).

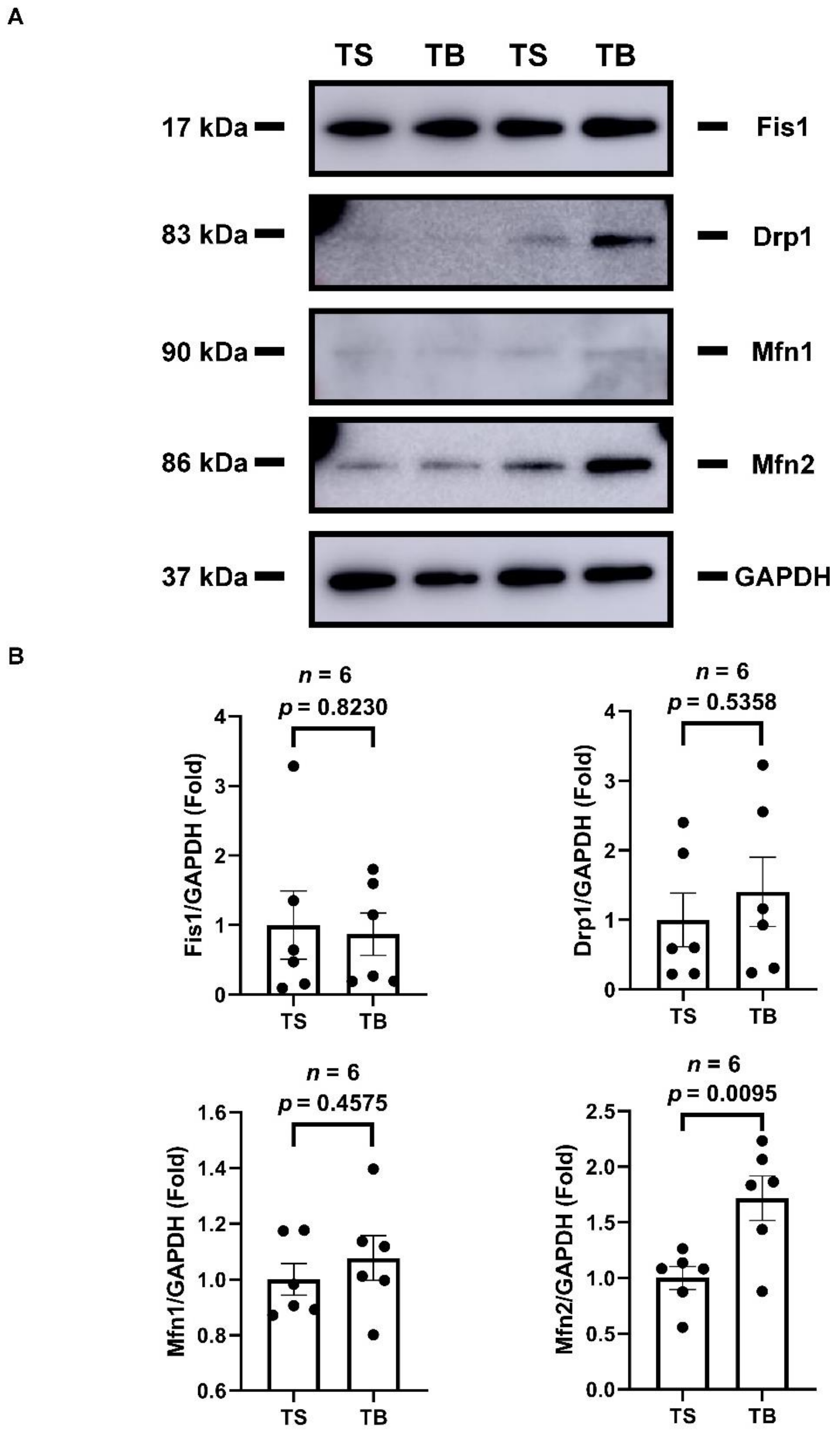

Figure S4. Mitochondrial fission and fusion protein expression in AMVMs. A-B, Representative western blot, and quantification of Fis1, Drp1, Mfn1, and Mfn2 levels in AMVMs. n = 6. TS vs. TB, Fis1, p = 0.8230 (NS). Drp1, p = 0.5358 (NS). Mfn1, p = 0.4575 (NS). Mfn2, p = 0.0095. TS, Tat-Scrambled; TB, Tat-Beclin 1; NS (not significant).

Expanded Materials & Methods

Animal studies

C57BL/6J mice were obtained from the Jackson lab.

The ATG7$^{F/F}$ mouse was provided by Dr. Massaki Komatsu[1], and the αMHC-merCremer mouse was obtained from Jackson lab[2]. All animal studies were conducted according to ethics guidelines provided by the Institutional Animal Care and Use Committee at University of Alabama at Birmingham. To induce MerCreMer (MCM) activity, tamoxifen (T5648, Sigma, Rockville, MD, USA) dissolved in peanut oil was administered intraperitoneally (IP, 20mg/kg per day x 5 days). Tissue- and induction-specific recombination was confirmed in αMHC-MCM lines by genomic DNA isolation (D4068, Zymo gDNA kit, Irvine, CA, USA) and PCR reaction around the target exon detailed in Dr. Masaki Komatsu's paper.

Primers that were used:
First Pair: HIND-FW: GGCTGCTACTTCTGCAATGATGT;
PST-RV: CAGGACAGAGACCATCAGCTCCAC; (WT 1500bp, Floxed 500bp).
Thermocycler conditions: cycle 1 (1X), 95.0ºC for 10 min; cycle 2 (35X), step 1 at 95.0ºC for 30 sec, step 2 at 62.0ºC for 30 sec; cycle 3 (1X), 72.0ºC for 10 min; then 4 ºC.
WT allele will produce a band at 1500bp and floxed allele will produce a 500bp band.

Second Pair: ATG7EX14F: TCTCCCAAGACAAGACAGGGTGAA;
ATG7EX14R: AAGCCAAAGGAAACCAAGGGAGTG. (WT 300bp, Floxed non band)
Thermocycler conditions: cycle 1 (1X), 95.0ºC for 10 min; cycle 2 (35X), step 1 at 95.0ºC for 30 sec, step 2 at 60.0ºC for 30 sec; cycle 3 (1X), 72.0ºC for 10 min; then 4 ºC.
WT allele will produce a band at 300bp and floxed allele will not produce a band.

Mouse model of time and myocardium-specific ATG7 knockout mice (ATG7F/F; αMHC-merCremer+) with tamoxifen injection, ATG7 cKO (KO)) were generated, and the loss of ATG7 was verified in isolated adult mouse ventricular myocytes (AMVMs). WT animals used in the αMHC-merCremer study were the cohort of ATG7F/F; αMHC-merCremer-, all of which were treated with the same tamoxifen regimen as the experimental group. We have previously shown that there is no difference between the cohorts of ATG7F/F; αMHC-merCremer- and WT αMHC-merCremer+ littermates treated with tamoxifen[3]. Animals were maintained in 12hr light/dark cycles with a standard chow diet ad libitum.

RNA purification and RT-qPCR

Snap-frozen tissues were disrupted in TRlzol (15596026, Fisher, Waltham, MA, USA) by bead beater (FastPrep-24, MP, Irvine, CA, USA) using disposable ceramic beads (6913-100, Fisher, Waltham, MA, USA) to extract total RNA. For isolated cells, TRIzol was used to extract total RNA. A total of 2000 ng of purified RNA was used for reverse-transcription reactions (4368814, Applied biosystems, Waltham, MA, USA). Quantitative PCR reactions were run with cDNA libraries in duplicate with SYBR Green master mix (Biorad, 1725125) on a BioRad CFX384 Real Time PCR machine. All of the primer sets were validated for doubling efficiency using cDNA standard curves. Quantification was performed using the ΔΔCt method to obtain relative fold change to WT or untreated sample in each sample set, normalized against a housekeeping gene (beta-actin for mouse). ATG7 primers that are used for mouse ATG7 expression are:

M-ATG7 311-330: TGGAGTTCAGTGCTTTTGAC,

M-ATG7 387-370: GGTGTTGTGCAGGGTTCC.

Internal control used is mouse beta-actin. The primers are:

mBAF: TCACCCACACTGTGCCCATCTACGA,

mBAR: CATCGGAACCGCTCGTTGCCAATAG.

Thermocycles: cycle 1 (1X), 95.0ºC for 1.5 min; cycle 2 (30X), step 1 at 95.0ºC for 20 sec, step 2 at 61.0ºC for 30 sec; cycle 3 (1X), 95.0ºC for 1 min; cycle 4 (1X), 55.0ºC for 1 min, cycle 5 (40X) 55.0ºC for 10 sec with an increase of 1.0ºC after each repeat for collecting melt curve data.

Western blotting

Snap-frozen tissue was resuspended in ice-cold lysis buffer (20 mM Tris-HCl pH=8.0, 150 mM NaCl, 0.1% TritonX-100, 2 mM EDTA, 1 mM DTT) with protease/phosphatase inhibitor cocktail (A32961, Thermo Scientific, Waltham, MA, USA). Tissues were processed using a Dounce homogenizer on ice. For tissue cultures, 1XSDS buffer was used to collect cells. Membranes were cut into strips to incubate different antibodies due to each antibody has a different exposure time. Primary antibodies used: LC3-II (rabbit anti-LC3 prepared in Hill Laboratory at UT Southwestern Medical Center, Dallas, TX, USA), GAPDH (10R-G109a, Fitzgerald, Acton, MA, USA), ATG7 (#2631, Cell Signaling Technology, Danvers, MA, USA), Fis1 (# PA5-22142, Invitrogen, Thermo Fisher Scientific, Waltham, MA, USA), Drp1 (ab184247, Abcam, Waltham, MA, USA), Mfn1 (sc-166644, Santa Cruz Biotechnology, Dallas, TX, USA), Mfn2 (ab50843, Abcam, Waltham, MA, USA). After incubation with secondary antibodies (NA931V, NA934V, GE healthcare, Chicago, IL, USA), membranes were imaged with an Amersham Imager 600 and quantified using ImageQuant software.

NRVM isolation

NRVMs were isolated using a Neonatal Cardiomyocyte Isolation kit (Neomyt Kit, NC6031, Cellutron, Baltimore, MD, USA) following the manufacturer’s protocol from 1 to 2-day old sprague dawley rats. After 16 hours, the cells were changed to 5% FBS containing medium (3:1 DMEM: M199, supplemented with L-Glutamine, penicillin/streptomycin, and BrdU). After 24 hours, it was changed to serum-free medium. The experiments were done 2-3 day after changing to serum-free medium.

AMVM isolation

The AMVM isolation was performed as previously described[4] with slight modifications using a Langendorff perfusion system. Briefly, the heart was cannulated through the aorta and perfused with perfusion buffer (in mM: 113 NaCl, 4.7 KCl, 0.6 KH2PO4, 0.6 Na2HPO4, 1.2 MgSO4, 12 NaHCO3, 10 KHCO3, 10 HEPES, 30 taurine, 10 BDM and 5.5 glucose) after isolating from the adult mouse, followed by digestion buffer (perfusion buffer supplemented with 300U/mL collagenase II and 1.5 mg/mL proteinase XIV). Then the heart was minced and filtered through a 100 µm-Cell strainer. The concentration of Ca^{2+} was gradually increased to a final concentration of 900 µM with repeated centrifugation and resuspension. Finally, the cells were resuspended and plated in the plating medium (DMEM 4.5 g/L glucose, supplemented with FBS, blebbistatin and penicillin/streptomycin) at 37°C and 5% CO2 for 1-3 hrs. Then the cells were cultured with DMEM 4.5 g/L glucose supplemented with BSA and blebbistatin. Cell purity was greater than 98% of cardiomyocytes. Detailed step by step protocol is available upon request.

Fluorescent microscope image acquisition and quantification

Dichlorofluorescein (H2DCFDA, MP36103, 10 µM, Invitrogen, Thermo Fisher Scientific, Waltham, MA, USA), tetramethylrhodamine methylester (TMRM, I34361, T668, 0.1 µM, Invitrogen, Thermo Fisher Scientific, Waltham, MA, USA), and MitoSOX Red (M36008, 5 µM, Invitrogen, Thermo Fisher

Scientific, Waltham, MA, USA) were used to measure total cellular ROS levels, mitochondrial membrane potential and mitochondrial ROS levels in 96-wells cultured NRVMs, respectively. After staining, cells were maintained in PBS and exposed with fixed exposure time at the same magnification using a Nikon Eclipse Ti fluorescent microscope. 5 fields of cells were quantified for each batch of images. Quantifications were using ImageJ software (1.53, NIH, Bethesda, MD, USA).

Assays of cell death

NRVMs cell death was detected by using MTT Assay Kit (V13154, 5 mg/mL, Invitrogen, Thermo Fisher Scientific, Waltham, MA, USA). NRVMs were treated with simulated ischemia/reperfusion (sI/R) of 5 hours ischemia and 3 hours of reperfusion, then changed to phenol red free culture medium (in mM: 130 NaCl, 4 KCl, 1.25 MgSO4, 1.2 CaCl2, 6.25 NaHCO3, 20 HEPLES, 20 D glucose) with 10% MTT solution. Incubated at 37ºC overnight then added SDS-HCl solution (SDS 0.1 g/ml, 0.01M HCl) the same volume of the culture medium to mix. Incubated at 37ºC for 4 hours then pipetted well to mix sample again, read the absorbance at 570 nm.

AMVMs were treated with simulated ischemia/reperfusion (sI/R) of 1 hour ischemia and 4 hours of reperfusion. Used a Nikon Eclipse Ti fluorescent microscope to take images at the same magnification in the bright field before sI/R, after 2 hours reperfusion, and after 4 hours reperfusion. Live cell numbers were counted by using ImageJ software (1.53, NIH, Bethesda, MD, USA).

Electron Microscope (EM)

Hearts tissues were fixed with 2% glutaraldehyde in 0.1 mol/L cacodylate buffer for 2 hours. Postfixation occurred in 2% osmium tetroxide in 0.1 mol/L cacodylate buffer and 1% aqueous uranyl acetate, each for 1 hour. An ascending series of ethanol washes (50%, 70%, 90%, 100%) was performed, followed by transitioning to propylene oxide and then a 1:1 mixture of propylene oxide and EMbed 812 (Electron Microscopy Sciences). The tissue was incubated in EMbed for 1 hour, then placed in a 70°C oven to polymerize. Sections (75–80 nm) were cut by using a Leica ultramicrotome and a Diatome diamond knife, collected on 200-mesh copper grids, and post-stained with 5% uranyl acetate in ethanol (10 minutes) and Reynold lead citrate (5 minutes). A Tecnai Spirit T12 transmission electron microscope, operating at 20 to 120 kV and equipped with a digital camera, was used to image the sections. Mitochondria numbers and size were analyzed by using ImageJ software (1.53, NIH, Bethesda, MD, USA).

ATP concentration measurement in AMVMs

AMVMs' ATP level was detected by using ATP Determination Kit (A22066, Invitrogen, Thermo Fisher Scientific, Waltham, MA, USA). AMVMs were treated with sI/R of 1 hour ischemia and 4 hours of reperfusion. Boiling water was used to inhibit ATPase5. AMVMs suspension was collected and centrifuged at 12000g for 5 mins at 4°C. The supernatant was used for bioluminescence measurement and read at 560 nm. The standard curve of ATP was obtained by serial dilutions of 10 nM ATP solution.

Oxygen consumption rate determination

Analyses of cellular bioenergetics were performed using the Seahorse XFe96 Extracellular Flux Analyzer (Agilent, Santa Clara, CA)[6, 7]. After isolation, the NRVMs were plated into an Agilent seahorse XF96 cell culture microplate (Product No.101085-004) at 28,000 cells/well for 4 days. On the day of the experiment the cells were exposed to 2 hours of ischemia as described above, then 2.5 µM TS/TB was added for a 4-hour reperfusion. After IR or normoxia the cells were changed into the XF media (non-buffered DMEM supplemented with 5.5 mM glucose, 1 mM pyruvate, and 4 mM glutamine, pH 7.36) at 37°C. Oxygen

consumption rate (OCR) was measured for basal OCR (OCR before oligomycin minus OCR after antimycin) followed by sequential injections of 1 µg/ml oligomycin, 1 µM FCCP, and 10 µM antimycin A. Mitochondrial parameters were calculated for ATP-linked (OCR before oligomycin minus OCR after oligomycin), proton leak (OCR after oligomycin minus OCR after antimycin), maximal (OCR after FCCP minus OCR after antimycin), reserve capacity (OCR after FCCP minus OCR before oligomycin), and non-mitochondrial (OCR after antimycin).

Statistical analysis

Data are expressed as the mean ± SEM. The sample size of each group was provided in individual figure legends and Table 2. All data and results were explicated in blinded fashion. GraphPad Prism software (version 8.0.2, GraphPad Software, San Diego, CA, USA) was used to perform statistical analyses. The normality of data was examined by Shapiro Wilk test before parametric or non-parametric tests. For comparisons, normally distributed data were analyzed by nonpaired 2 tailed Student t-test (two-group analysis) and two-way ANOVA analysis followed by Tukey's post hoc test (multiple group analysis). Paired data were analyzed by one-way ANOVA represents matched analysis followed by Tukey's post hoc multi comparison test (multiple group analysis). No experiment wide/across test multiple test correction was applied and only within test corrections were made. The representative image was selected from one of the repeated experiments that best matched the mean value. Detailed statistical analysis information including normalization procedures, sample sizes, and named statistical tests for all main and supplementary figures is described in Table 2 in the Supplementary Material.

Table S1 Sequences of forward and reverse primers used for PCR

species	Gene	Primer	Primer Sequence (5´-3`)
Rat	Long Mito 13593	Forward	CCCAGCCACCACTATCATTCAAGTAG
Rat	Long Mito 13364	Reverse	TAGAGTTTTTTGAGGAATAATTCGGTG
Rat	Short Mito 13278	Forward	CCCACCAAACTATCATCATTCTCAAC
Rat	Short Mito 13364	Reverse	TAGAGTTTTTTGAGGAATAATTCGGTG
Mouse	COXII	Forward	CCATCCCAGGCCGACTAA
Mouse	COXII	Reverse	CAGAGCATTGGCCATAGAATAACC
Mouse	ATP synthase 6	Forward	CAAACAAATAATGCTAATCCACACACC
Mouse	ATP synthase 6	Reverse	GCTGTAAGCCGGACTGCTAATG
Mouse	Beta-actin	Forward	TCACCCACACTGTGCCCATCTACGA
Mouse	Beta-actin	Reverse	CATCGGAACCGCTCGTTGCCAATAG
Rat	COXII	Forward	CAATCCCCGGCCGCCTAA
Rat	COXII	Reverse	CAGAGCATTGGCCATAGAATAGAC
Rat	ATP synthase 6	Forward	CAAACAAATAATGTTAATCCACACACC
Rat	ATP synthase 6	Reverse	GCTGTTAGTCGTACTGCTAGTG
Rat	Beta-actin	Forward	TCACCCACACTGTGCCCATCTATGA
Rat	Beta-actin	Reverse	CATCGGAACCGCTCATTGCCGATAG
Mouse	PGC-1α	Forward	AGCCGTGACCACTGACAACGAG
Mouse	PGC-1α	Reverse	GCTGCATGGTTCTGAGTGCTAAG
Mouse	Drp1	Forward	GGAACCAACAACAGGCAACT
Mouse	Drp1	Reverse	GCAACTGGAACTGGCACAT
Mouse	Fis1	Forward	AAGTATGTGCGAGGGCTGTT
Mouse	Fis1	Reverse	AGCCAGTCCAATGAGTCCAG
Mouse	Opa1	Forward	ATCCTAACGCCATCATCCTG

Mouse	Opa1	Reverse	GTTGTATCCTGCTTGGACTGG
Mouse	Mfn1	Forward	TCAGAGCCCATCTTTCAGGT
Mouse	Mfn1	Reverse	GTTTCCAGCCCACTGTTTTC
Mouse	Mfn2	Forward	CTCCATCAGGACGAGCAGTT
Mouse	Mfn2	Reverse	GCACAAACACATCAGCATCC
Mouse	ATG7	Forward	TGGAGTTCAGTGCTTTTGAC
Mouse	ATG7	Reverse	GGTGTTGTGCAGGGTTCC
Mouse	PARIS	Forward	AGTTGGACTCTGGAGCAGGA
Mouse	PARIS	Reverse	GCTGCTGTGTTGAGCTTCAG
Mouse	Nrf2	Forward	CTGAACTCCTGGACGGGACTA
Mouse	Nrf2	Reverse	CGGTGGGTCTCCGTAAATGG

Table S2 Detailed statistical analysis information for all main and supplementary figures

Figure	Groups (Sample size)	Statistical analysis	Normalization	P value
1B	Normoxia TS (n=3)	2-way-ANOVA analysis followed by Tukey's post-hoc multi-comparison	Normalized to the average of Normoxia TS	
	Normoxia TB (n=3)			
	I2R4 TS (n=3)			
	I2R4 TB (n=3)			
	Normoxia TS BFA (n=3)			
	Normoxia TB BFA (n=3)			
	I2R4 TS BFA (n=3)			

	I2R4 TB BFA (n=3)			P=0.0085 *vs* I2R4 TS BFA
1D	Normoxia TS (n=4)	2-way-ANOVA analysis followed by Tukey's post-hoc multi-comparison	Normalized to the individual of Normoxia TS	
	Normoxia TB (n=4)			
	I2R4 TS (n=4)			P=0.0066 *vs* Normoxia TS
	I2R4 TB (n=4)			P=0.0223 *vs* I2R4 TS
1F	Normoxia TS (n=4)	2-way-ANOVA analysis followed by Tukey's post-hoc multi-comparison	Normalized to the individual of Normoxia TS	
	Normoxia TB (n=4)			
	I2R4 TS (n=4)			P=0.0025 *vs* Normoxia TS
	I2R4 TB (n=4)			P=0.0201 *vs* I2R4 TS
2B	Normoxia TS (n=3)	2-way-ANOVA analysis followed by Tukey's post-hoc multi-comparison	Normalized to the individual of Normoxia TS	
	Normoxia TB (n=3)			
	I2R6 TS (n=3)			P=0.024 *vs* Normoxia TS
	I2R6 TB (n=3)			P=0.0022 *vs* I2R6 TS
2C	Normoxia TS (n=3)	2-way-ANOVA analysis followed by Tukey's post-hoc multi-comparison	Normalized to the individual of Normoxia TS	
	Normoxia TB (n=3)			
	I2R6 TS (n=3)			NS *vs* Normoxia TS
	I2R6 TB (n=3)			P=0.0117 *vs* I2R6 TS
2D	Normoxia TS (n=3)	2-way-ANOVA analysis followed by Tukey's post-hoc multi-comparison	Normalized to the individual of Normoxia TS	
	Normoxia TB (n=3)			
	I2R6 TS (n=3)			NS *vs* Normoxia TS

	I2R6 TB (n=3)			P=0.0009 *vs* I2R6 TS
2F	Normoxia TS (n=4)	2-way-ANOVA analysis followed by Tukey's post-hoc multi-comparison	Normalized to the individual of Normoxia TS	
	Normoxia TB (n=4)			
	I2R4 TS (n=4)			P=0.0001 *vs* Normoxia TS
	I2R4 TB (n=4)			P=0.001 *vs* I2R4 TS
2H	Normoxia TS (n=5)	1-way-ANOVA analysis represents matched followed by Tukey's post-hoc multi-comparison	Normalized to the individual of Normoxia TS	
	Normoxia TB (n=5)			
	I2R4 TS (n=5)			
	I2R4 TB (n=5)			NS *vs* Other three groups
2I	Normoxia TS (n=5)	1-way-ANOVA analysis represents matched followed by Tukey's post-hoc multi-comparison	Normalized to the individual of Normoxia TS	
	Normoxia TB (n=5)			
	I2R4 TS (n=5)			
	I2R4 TB (n=5)			NS *vs* Other three groups
2J	Normoxia TS (n=5)	1-way-ANOVA analysis represents matched followed by Tukey's post-hoc multi-comparison	Normalized to the individual of Normoxia TS	
	Normoxia TB (n=5)			
	I2R4 TS (n=5)			
	I2R4 TB (n=5)			NS *vs* Other three groups
2K	Normoxia TS (n=5)	1-way-ANOVA analysis represents matched followed by Tukey's post-hoc multi-comparison	Normalized to the individual of Normoxia TS	
	Normoxia TB (n=5)			
	I2R4 TS (n=5)			P=0.0448 *vs* Normoxia TS
	I2R4 TB (n=5)			P=0.0009 *vs* I2R4 TS
2L	Normoxia TS (n=5)			

	Normoxia TB (n=5)	1-way-ANOVA analysis represents matched followed by Tukey’s post-hoc multi-comparison	Normalized to the individual of Normoxia TS	
	I2R4 TS (n=5)			P=0.0217 *vs* Normoxia TS
	I2R4 TB (n=5)			P=0.0097 *vs* I2R4 TS
3B	TS I (n=5)	2-way-ANOVA analysis followed by Tukey’s post-hoc multi-comparison	Normalized to the average of TS R	
	TS B (n=5)			
	TS R (n=5)			
	TB I (n=5)			
	TB B (n=5)			P=0.0054 *vs* TS B
	TB R (n=5)			
3C	TS I (n=6)	2-way-ANOVA analysis followed by Tukey’s post-hoc multi-comparison	Normalized to the average of TS R	
	TS B (n=6)			
	TS R (n=6)			
	TB I (n=6)			
	TB B (n=6)			P=0.0363 *vs* TS B
	TB R (n=6)			
3D	TS I (n=6)	2-way-ANOVA analysis followed by Tukey’s post-hoc multi-comparison	Normalized to the average of TS R	
	TS B (n=6)			
	TS R (n=6)			
	TB I (n=6)			
	TB B (n=6)			P=0.0394 *vs* TS B
	TB R (n=6)			
3E	TS I (n=5)	2-way-ANOVA analysis followed	Normalized to the average of TS R	
	TS B (n=5)			

	TS R (n=5)	by Tukey's post-hoc multi-comparison		
	TB I (n=5)			
	TB B (n=5)			P=0.0311 *vs* TS B
	TB R (n=5)			
3F	TS I (n=7)	2-way-ANOVA analysis followed by Tukey's post-hoc multi-comparison	Normalized to the average of TS R	
	TS B (n=7)			
	TS R (n=7)			
	TB I (n=6)			
	TB B (n=6)			P=0.0292 *vs* TS B
	TB R (n=6)			
3G	TS I (n=7)	2-way-ANOVA analysis followed by Tukey's post-hoc multi-comparison	Normalized to the average of TS R	
	TS B (n=7)			
	TS R (n=7)			
	TB I (n=6)			
	TB B (n=6)			P=0.3763 *vs* TS B
	TB R (n=6)			
3H	TS I (n=7)	2-way-ANOVA analysis followed by Tukey's post-hoc multi-comparison	Normalized to the average of TS R	
	TS B (n=7)			
	TS R (n=7)			
	TB I (n=6)			
	TB B (n=6)			P=0.0091 *vs* TS B
	TB R (n=6)			
3I	TS I (n=7)	2-way-ANOVA analysis followed	Normalized to the average of TS R	
	TS B (n=7)			

	TS R (n=7)	by Tukey's post-hoc multi-comparison		
	TB I (n=6)			
	TB B (n=6)			P=0.0444 *vs* TS B
	TB R (n=6)			
3J	TS I (n=7)	2-way-ANOVA analysis followed by Tukey's post-hoc multi-comparison	Normalized to the average of TS R	
	TS B (n=7)			
	TS R (n=7)			
	TB I (n=6)			
	TB B (n=6)			P=0.0323 *vs* TS B
	TB R (n=6)			
3L Left	TS (n=4)	Nonpaired 2-tailed Student t test	Normalized to the average of TS	
	TB (n=4)			NS *vs* TS
3L Right	TS (n=4)	Nonpaired 2-tailed Student t test	Normalized to the average of TS	
	TB (n=4)			P=0.0282 *vs* TS
4B Left	WT (n=3)	Nonpaired 2-tailed Student t test	Normalized to the average of WT	
	KO (n=3)			P=0.0151 *vs* WT
4B Right	WT (n=3)	Nonpaired 2-tailed Student t test	Normalized to the average of WT	
	KO (n=3)			P=0.002 *vs* WT
4C	WT (n=6)	Nonpaired 2-tailed Student t test	Normalized to the average of WT	
	KO (n=5)			P=0.0073 *vs* WT
4D	WT TS (n=6)	2-way-ANOVA analysis followed by Tukey's post-hoc multi-comparison	Normalized to the average of WT TS	
	WT TB (n=6)			P=0.0212 *vs* WT TS
	KO TS (n=5)			NS *vs* WT TS

	KO TB (n=5)			NS *vs* KO TS
4E	WT TS (n=6)	2-way-ANOVA analysis followed by Tukey's post-hoc multi-comparison	Normalized to the average of WT TS	
	WT TB (n=6)			P=0.048 *vs* WT TS
	KO TS (n=5)			NS *vs* WT TS
	KO TB (n=5)			NS *vs* KO TS
4F	WT TS (n=6)	2-way-ANOVA analysis followed by Tukey's post-hoc multi-comparison	Normalized to the average of WT TS	
	WT TB (n=6)			P=0.0365 *vs* WT TS
	KO TS (n=5)			NS *vs* WT TS
	KO TB (n=5)			NS *vs* KO TS
4G	WT TS (n=6)	2-way-ANOVA analysis followed by Tukey's post-hoc multi-comparison	Normalized to the average of WT TS	
	WT TB (n=6)			P=0.3713 *vs* WT TS
	KO TS (n=5)			NS *vs* WT TS
	KO TB (n=5)			NS *vs* KO TS
4H	WT TS (n=6)	2-way-ANOVA analysis followed by Tukey's post-hoc multi-comparison	Normalized to the average of WT TS	
	WT TB (n=6)			P=0.0104 *vs* WT TS
	KO TS (n=5)			NS *vs* WT TS
	KO TB (n=5)			NS *vs* KO TS
4I	WT TS (n=6)	2-way-ANOVA analysis followed by Tukey's post-hoc multi-comparison	Normalized to the average of WT TS	
	WT TB (n=6)			P=0.0243 *vs* WT TS
	KO TS (n=5)			NS *vs* WT TS
	KO TB (n=5)			NS *vs* KO TS
4J	WT TS (n=6)	2-way-ANOVA analysis followed by Tukey's post-hoc multi-comparison	Normalized to the average of WT TS	
	WT TB (n=6)			P=0.0494 *vs* WT TS

	KO TS (n=5)			NS *vs* WT TS
	KO TB (n=5)			NS *vs* KO TS
5A	WT (n=10)	Nonpaired 2-tailed Student t test	Normalized to the average of WT	
	KO (n=9)			P=0.0002 *vs* WT
5B	WT TS (n=6)	2-way-ANOVA analysis followed by Tukey's post-hoc multi-comparison	Normalized to the average of WT TS	
	WT TB (n=4)			P=0.023 *vs* WT TS
	KO TS (n=5)			NS *vs* WT TS
	KO TB (n=4)			NS *vs* KO TS
5C	WT TS (n=6)	2-way-ANOVA analysis followed by Tukey's post-hoc multi-comparison	Normalized to the average of WT TS	
	WT TB (n=4)			P=0.0421 *vs* WT TS
	KO TS (n=5)			NS *vs* WT TS
	KO TB (n=4)			NS *vs* KO TS
5D	WT TS (n=6)	2-way-ANOVA analysis followed by Tukey's post-hoc multi-comparison	Normalized to the average of WT TS	
	WT TB (n=4)			P=0.0216 *vs* WT TS
	KO TS (n=5)			NS *vs* WT TS
	KO TB (n=4)			NS *vs* KO TS
5E	WT TS (n=6)	2-way-ANOVA analysis followed by Tukey's post-hoc multi-comparison	Normalized to the average of WT TS	
	WT TB (n=4)			P=0.0205 *vs* WT TS
	KO TS (n=5)			NS *vs* WT TS
	KO TB (n=4)			NS *vs* KO TS
5F	WT TS (n=6)	2-way-ANOVA analysis followed by Tukey's post-hoc multi-comparison	Normalized to the average of WT TS	
	WT TB (n=4)			P=0.0121 *vs* WT TS
	KO TS (n=5)			NS *vs* WT TS

	KO TB (n=4)			NS *vs* KO TS
5G	WT TS (n=6)	2-way-ANOVA analysis followed by Tukey's post-hoc multi-comparison	Normalized to the average of WT TS	
	WT TB (n=4)			P=0.0459 *vs* WT TS
	KO TS (n=5)			NS *vs* WT TS
	KO TB (n=4)			NS *vs* KO TS
5H	WT TS (n=6)	2-way-ANOVA analysis followed by Tukey's post-hoc multi-comparison	Normalized to the average of WT TS	
	WT TB (n=4)			P=0.0095 *vs* WT TS
	KO TS (n=5)			NS *vs* WT TS
	KO TB (n=4)			NS *vs* KO TS
S1B Up	TS (n=4)	Nonpaired 2-tailed Student t test	Normalized to the average of TS	
	TB (n=4)			NS *vs* TS
S1B Down	TS (n=4)	Nonpaired 2-tailed Student t test	Normalized to the average of TS	
	TB (n=4)			P=0.0071 *vs* TS
S1C	Normoxia TS (n=6)	2-way-ANOVA analysis followed by Tukey's post-hoc multi-comparison	Normalized to the average of Normoxia TS	
	Normoxia TB (n=6)			P=0.0048 *vs* Normoxia TS
	I1R4 TS (n=6)			NS *vs* Normoxia TS
	I1R4 TB (n=6)			P=0.0278 *vs* I1R4 TS
S1D	Normoxia TS (n=4)	2-way-ANOVA analysis followed by Tukey's post-hoc multi-comparison	Normalized to the average of Normoxia TS	
	Normoxia TB (n=4)			
	I2R4 TS (n=4)			P<0.0001 *vs* Normoxia TS
	I2R4 TB (n=4)			P=0.0282 *vs* I2R4 TS
S2A Up Left	TS (n=4)			

	TB (n=4)	Nonpaired 2-tailed Student t test	Normalized to the average of TS	NS *vs* TS
S2A Up Right	TS (n=4)	Nonpaired 2-tailed Student t test	Normalized to the average of TS	
	TB (n=4)			NS *vs* TS
S2A Down Left	TS (n=4)	Nonpaired 2-tailed Student t test	Normalized to the average of TS	
	TB (n=4)			NS *vs* TS
S2A Down Right	TS (n=4)	Nonpaired 2-tailed Student t test	Normalized to the average of TS	
	TB (n=4)			NS *vs* TS
S3A	WT TS (n=6)	2-way-ANOVA analysis followed by Tukey's post-hoc multi-comparison	Normalized to the average of WT TS	
	WT TB (n=6)			P=0.0212 *vs* WT TS
	KO TS (n=5)			NS *vs* WT TS
	KO TB (n=5)			NS *vs* KO TS
S3B Left	WT TS (n=6)	2-way-ANOVA analysis followed by Tukey's post-hoc multi-comparison	Normalized to the average of WT TS	
	WT TB (n=6)			NS *vs* Other three groups
	KO TS (n=5)			
	KO TB (n=5)			
S3B Right	WT TS (n=6)	2-way-ANOVA analysis followed by Tukey's post-hoc multi-comparison	Normalized to the average of WT TS	
	WT TB (n=6)			NS *vs* Other three groups
	KO TS (n=5)			
	KO TB (n=5)			
S4B Up Left	TS (n=6)	Nonpaired 2-tailed Student t test	Normalized to the average of TS	
	TB (n=6)			NS *vs* TS
S4B Up Right	TS (n=6)	Nonpaired 2-tailed Student t test	Normalized to the average of TS	
	TB (n=6)			NS *vs* TS
S4B Down Left	TS (n=6)			

	TB (n=6)	Nonpaired 2-tailed Student t test	Normalized to the average of TS	NS *vs* TS
S4B Down Right	TS (n=6)	Nonpaired 2-tailed Student t test	Normalized to the average of TS	
	TB (n=6)			P=0.0095 *vs* TS

1. Komatsu M, Waguri S, Ueno T, Iwata J, Murata S, Tanida I, Ezaki J, Mizushima N, Ohsumi Y, Uchiyama Y, Kominami E, Tanaka K and Chiba T. Impairment of starvation-induced and constitutive autophagy in Atg7-deficient mice. *The Journal of cell biology*. 2005;169:425-34.

2. Sohal DS, Nghiem M, Crackower MA, Witt SA, Kimball TR, Tymitz KM, Penninger JM and Molkentin JD. Temporally regulated and tissue-specific gene manipulations in the adult and embryonic heart using a tamoxifen-inducible Cre protein. *Circ Res*. 2001;89:20-5.

3. Xie M, Cho GW, Kong Y, Li DL, Altamirano F, Luo X, Morales CR, Jiang N, Schiattarella GG, May HI, Medina J, Shelton JM, Ferdous A, Gillette TG and Hill JA. Activation of Autophagic Flux Blunts Cardiac Ischemia/Reperfusion Injury. *Circulation research*. 2021;129:435-450.

4. Li D, Wu J, Bai Y, Zhao X and Liu L. Isolation and culture of adult mouse cardiomyocytes for cell signaling and in vitro cardiac hypertrophy. *J Vis Exp*. 2014.

5. Yang NC, Ho WM, Chen YH and Hu ML. A convenient one-step extraction of cellular ATP using boiling water for the luciferin-luciferase assay of ATP. *Anal Biochem*. 2002;306:323-7.

6. Dranka BP, Benavides GA, Diers AR, Giordano S, Zelickson BR, Reily C, Zou L, Chatham JC, Hill BG, Zhang J, Landar A and Darley-Usmar VM. Assessing bioenergetic function in response to oxidative stress by metabolic profiling. *Free Radic Biol Med*. 2011;51:1621-35.

7. Redmann M, Benavides GA, Wani WY, Berryhill TF, Ouyang X, Johnson MS, Ravi S, Mitra K, Barnes S, Darley-Usmar VM and Zhang J. Methods for assessing mitochondrial quality control mechanisms and cellular consequences in cell culture. *Redox Biol*. 2018;17:59-69.
